# Primitive neuroectodermal tumor of urinary bladder: A case report and literature review

**DOI:** 10.1097/MD.0000000000023032

**Published:** 2020-11-06

**Authors:** Liang Gao, Wenjie Xie, Kun Li, Gaomin Huang, Yuanhai Ji, Yangkang Ou, Jie Chen

**Affiliations:** a Department of Urology, Jiangxi Provincial People's Hospital Affiliated to Nanchang University; b Department of Urology, the First Affiliated Hospital of Nanchang University; c Department of Urology, Jiujiang No.1 People's Hospital.

**Keywords:** CD99, laparoscopic radical cystectomy, primitive neuroectodermal tumor, transurethral resection of bladder tumor, urinary bladder tumor

## Abstract

**Rationale:**

Primitive neuroectodermal tumor (PNET) of the urinary bladder is a highly aggressive tumor with high local recurrence and distant metastasis rates in cases of incomplete excision. We report a case of a young female patient, in whom early laparoscopic radical cystectomy combined with standard lymph node dissection and a modified vincristine, doxorubicin hydrochloride, and cyclophosphamide (VAC) chemotherapy regimen was controversial. Because PNET of the urinary bladder is a rare malignancy, the standard treatment regimen has not yet been established. It is not clear whether surgery combined with postoperative chemotherapy for PNET patients may be superior to surgery alone on long term survival.

**Patient concerns:**

The patient was a 45-year-old Chinese woman who complained of lower urinary tract symptoms, including urgency, frequency, and difficulty in urination, for 2 months.

**Diagnoses:**

PNET.

**Interventions:**

The patient underwent laparoscopic radical cystectomy and standard lymph node dissection, combined with modified VAC chemotherapy regimens.

**Outcomes:**

After undergoing radical surgery in 2018, the patient completed 6 courses of adjuvant chemotherapy. Abdominal and thorax computed tomography scanning was performed 3, 6, 9, and 12 months after the surgery was completely free of tumor. The patient is still alive with no signs of recurrent disease 2 years after diagnosis.

**Lessons:**

Radical surgery and standard lymphadenectomy combined with adjuvant chemotherapy may be essential to improve the prognosis of PNET of the urinary bladder.

## 1 Introduction

Primitive neuroectodermal tumors (PNETs) are malignant, small, round-cell tumors characterized by neuroectodermal differentiation that occurs predominantly in the bones and soft tissue of children and young adults. The peak incidence of PNET is at ages from 10 to 20 years.^[1]^ On the contrary, PNET of the urinary bladder is extremely rare, but more frequent in older adults. Most of the patients in the only 18 reported cases identified in the medical literature were at a late stage when diagnosed with the disease, and the tumor had already infiltrated the muscle or metastasized, leading to poor prognosis.

Here, we report a case of a newly diagnosed bladder tumor. The complete clinical, pathological, and follow-up data of the patient were used to confirm PNET of the urinary bladder. Written informed consent for the investigation and the publication of the case report was obtained from the patient. This study was approved by the ethical review committee of the First Affiliated Hospital of Nanchang University (Nanchang, Jiangxi, China).

## 2 Case report

**Figure 1:**
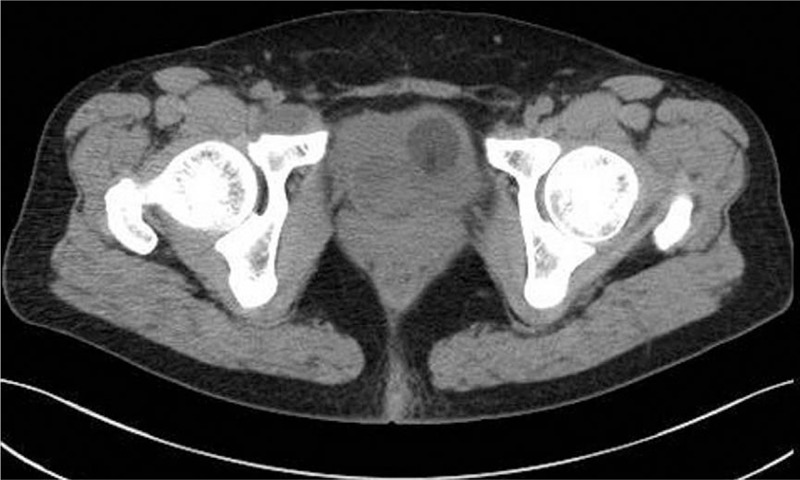
Pelvic computed tomography (CT) revealed a rounded soft-tissue mass (size of about 3.0 × 3.0 cm^2^) with a homogeneously hypodense shadow on the wall of the urinary bladder on the right side, and no abnormal pelvic lymph nodes were observed.

**Figure 2:**
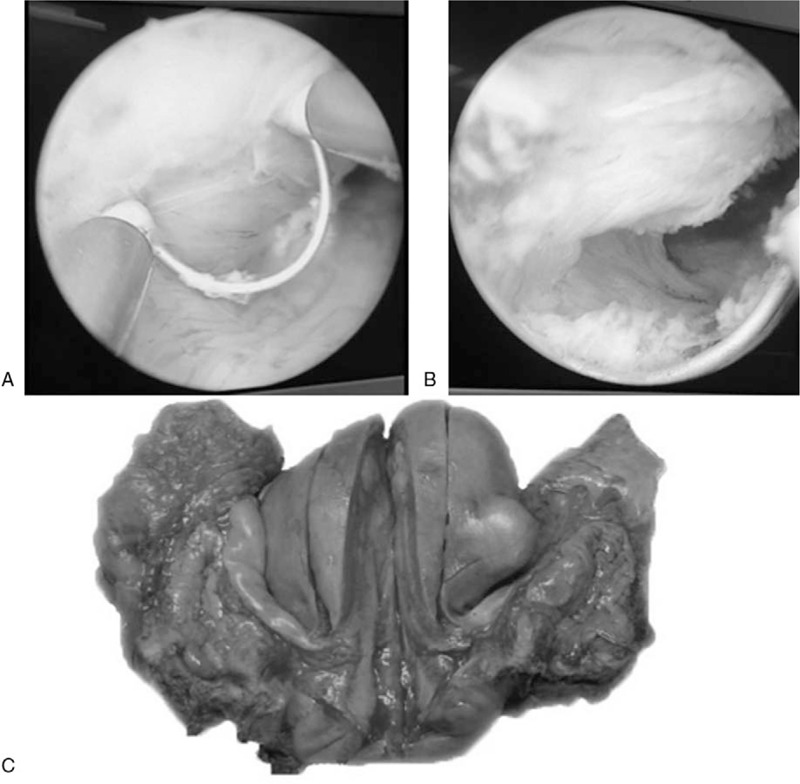
(A and B) First intraoperative exploration revealed a smooth, rounded soft-tissue mass with an approximate diameter of 3.0 cm, located on the right-side wall of the bladder neck. (C) A specimen of PNET after radical cystectomy still limited to the urinary bladder.

**Figure 3:**
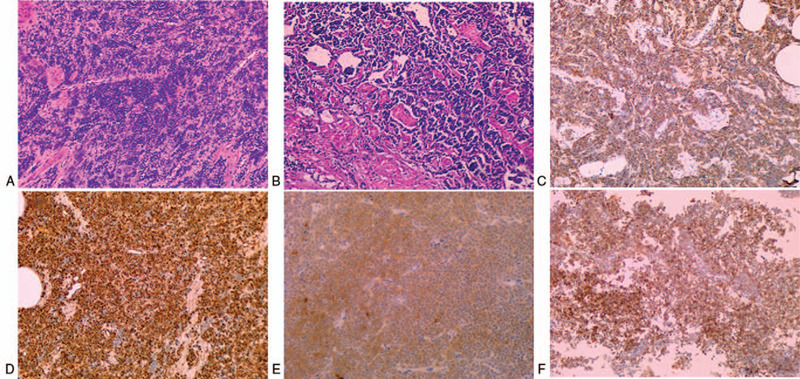
(A) Histological examination revealed a large number of malignant, small, round blue cells arranged in sheet- and nest-shaped patterns. (B) At high magnification, the neoplastic cells crowded the hyperchromatic nuclei (A: Hematoxylin and eosin (H&E) original magnification × 100; B: H&E original magnification × 200). Immunohistochemical results showed that tumor cells had significant immunoreactivity to (C) CD99, (D) vimentin, (E) synaptophysin, and (F) CD56 antigen (C–F: Original magnification × 200).

In September 2018, a 45-year-old Chinese woman with urgency, frequency, and dysuria for 2 months was admitted to the department. No gross hematuria and weight loss were registered. She had an unremarkable medical history, but the patient's mother had been diagnosed with breast cancer in a physical examination a year ago. An emergency indwelling balloon urethral catheter was inserted due to acute urinary retention. During initial admission, cystoscopy revealed a smooth, rounded, soft-tissue mass with an approximate diameter of 3.0 cm, located on the right-side wall of the bladder neck, without fluffy and cauliflower-like necrotic structures, which led to bladder outlet obstruction. No cyst biopsy was performed. Pelvic computed tomography (CT) revealed a rounded soft-tissue mass with a homogeneous hypodense shadow (approximate size 3.0 × 3.0 cm^2^) on the right side of the urinary bladder wall. No abnormal pelvic lymph nodes were observed (Fig. 1). No metastases were detected by thoracic CT and single-photon emission CT radionuclide bone scanning in distant visceral organs or bones. Physical examination, urinary cytology, and routine blood and biochemical indexes were normal. Transurethral resection of the bladder tumor (TURBT) was performed on the 5th day after hospitalization (Fig. 2). Postoperative microscopic examinations revealed invasive undifferentiated carcinoma originating from the bladder. The tumor included the mucosa, submucosa, and the muscular layer and consisted of sheets and nests of densely packed uniform, small, and round blue cells with hyperchromatic nuclei, and little cytoplasm. Rosette or pseudorosette structures were not visible, but conspicuous mitotic activity was noted (Fig. 3). The tumor cells showed significant immunoreactivity to cluster of differentiation 99 (CD99). However, they were negative for cytokeratin 8, cytokeratin 18, cytokeratin 20, and epithelial membrane antigen, while they should have been cytokeratin positive in the case of adenocarcinoma (Fig. 3). Thus, further immunohistochemical or molecular analysis was indispensable to confirm the diagnosis. This patient showed obvious immunoreactivity to vimentin, synaptophysin, cluster of differentiation 56 (CD56), and neuron-specific enolase. These results were suggestive of diagnosis but were not necessarily apparent pathognomonic signs (Fig. 3). Staining for desmin, myogenic regulatory protein-1, myogenin, endothelial transcription factor-3, leukocyte common antigen, paired box protein-8, S100, thyroid transcription factor-1, human melanoma black-45, periodic acid–Schiff stain, and periodic acid–silver methenamine was negative. Finally, the pathological examination confirmed the diagnosis of PNET of the bladder.

The patient underwent secondary electrotomy again, within 1 month after the first TURBT and laparoscopic radical cystectomy with an ileal conduit, total hysterectomy, and standard pelvic lymphadenectomy in December 2018 (Fig. 2). Combination treatment, involving modified VAC chemotherapy regimens and liposome drug release of doxorubicin hydrochloride (42 mg on day 1) and cyclophosphamide (1200 mg on day 1), was given for 1 month postoperatively. This therapy was repeated every 3 weeks, with alternating courses of liposome release of VAC (2.0 mg on day 1). After 6 cycles of adjuvant chemotherapy, CT scan of the chest and abdomen was performed 3, 6, 9, and 12 months after the radical surgery, which revealed no signs of any recidivation or metastasis.

## 3 Literature review and discussion

PNET is a distinct high-grade malignant neoplasm belonging to the Ewing family of tumors and originating from embryonic migrating cells of the neural crest, which occurs predominantly in the bones and soft tissue of children and adolescents.^[1]^ PNET was first discovered in orthopedic patients as a relatively infrequent, highly aggressive malignancy. The tumor originated from the brain or around the spines and was then most commonly located in the hips, shoulders, and extremities, but rarely occurred in parenchymatous organs.^[2]^ Later, Rao et al^[3]^ considered that PNET of the bladder was a tumor with aggressive behavior in children and young adults. So far, however, only 18 cases of PNET of the bladder have been reported in the published literature, of which only 6 patients underwent radical cystectomy. The patients aged from 10 to 81 years, with a mean age of approximately 42 years. Nonetheless, most patients (63%) were aged more than 30 years, including the patient in the present case study. The susceptible population of PNET of the urinary bladder may differ from that of the Ewing sarcoma, which mainly occurs in young people. The former is usually found in the elderly people. The most common previously reported symptoms were hematuria (68.40%), dysuria (42.10%), frequency (15.70%), and hydronephrosis (15.70%). In the patient in the present study, the cardinal clinical symptoms were dysuria with urinary irritation rather than hematuria, which might have been caused by the highly invasive and rapid growth of the tumor, which had become sufficiently large to press against bladder outlet and interfere with urination.

Recent evidence showed that immunodeficiency might be a risk factor for PNET of the bladder.^[4]^ A review of the literature revealed that out of 5 (28%) patients who had immunodeficiency before the diagnosis of PNET of the urinary bladder, 1 patient received immunosuppressive drugs after renal transplantation and 4 patients underwent chemotherapy for other malignant tumors. Molecular tests by fluorescence in situ hybridization (FISH) or reverse transcriptase–polymerase chain reaction (RT-PCR) revealed that the Ewing sarcoma/friend leukemia integration-1 (*EWS/FLI-1*) fusion gene was detected in some immunodeficient patients with PNET.^[5,6]^*EWS/FLI-I* fusion proteins were reportedly generated by the chromosomal translocation of t(11; 22)(q24; q12), which might be one of the pathogenetic factors of PNET.^[7]^ However, *EWS/FLI-I* fusion proteins were detected in 10 patients with PNET of the bladder (91%) without immunodeficiency. As a result, the relationship between immunodeficiency and PNET of the bladder and the precise pathogenesis were still not explicit, and hence, larger-sample studies on this association are needed to further expound the specific underlying mechanism accurately.

Tumor local aggression and distant metastasis could be the main cause of poor prognosis. In a previous series, 7 of 18 patients (38.9%) had regional or distant metastasis, among which 28.6% had pelvic or retroperitoneal lymph tissue, 28.6% had secondary infiltration or metastasis to the ileum and rectum, and 42.9% had lung metastases.^[8–15]^ In addition, of the 7 patients with metastasis, 4 (57.1%) died, with a mean postoperative survival period of 7.8 months (0.5–22 months).

Generally, the initial diagnosis of PNET of the bladder is made on the basis of the presence of small, round-cell malignant tumors. However, further immunohistochemistry or molecular analyses are required to more precisely confirm the diagnosis. In previous studies, light microscope examination found a relatively rare formation of rosettes or pseudorosettes, which was observed only in 4 cases.^[6,8,16]^ In the immunohistochemical analysis, CD99 protein was positively expressed. Despite not being considered a specific biomarker for PNET of the bladder, CD99 was nearly always present in these tumors. In addition, the positive expression rate of S-100, synaptophysin, vimentin, neuron-specific enolase, and desmin in PNET of the bladder was 83.3%, 58.3%, 70%, 33.3%, and 18.7%, respectively. It was speculated that rosettes or pseudorosettes combined with more than 2 specific immunohistochemical marker detection are an important clue in the clinical diagnosis of this disease.^[17]^ Currently, in addition to traditional histology and immunohistochemistry, the diagnosis of PNET of the bladder was made in combination with FISH or RT-PCR.^[18,19]^ Previously, the presence of *EWS/FLI-1* fusion gene was detected in 92% of the cases using new assistive technologies.^[13]^ In the patient discussed in the present study, a strong and diffuse immunoreactivity to the CD99 antigen was observed, especially for vimentin, synaptophysin, neuron-specific enolase, and CD56 antigen. A series of immunohistochemical results may be more helpful in the diagnosis of PNET of the bladder. Contrary to the findings of most reports, the immunoreactivity for S100 was negative in the present case, Ewing translocation gene testing was not performed.

Aggressive surgery is still the main comprehensive therapeutic method of the bladder. Radical cystectomy was performed in 6 of 18 cases (33.3%), lung metastasis was detected in 1 of 6 cases; of these, 5 patients received postoperative chemotherapy, and the treatment of the last one was not reported.^[4,11,20–23]^ Of the 6 patients who underwent combined therapy, 1 patient (17%) died 14 months after the surgery, and the mean survival period after the surgery was at least 22.5 months (14–36 months). TURBT or partial cystectomy was performed in 11 of the remaining 12 cases (91.7%),^[3,6,8–10,12,15,16,24,25]^ pelvic or retroperitoneal lymph node metastasis was detected in 5 of 11 cases, and the treatment of the last one was not reported (Table 1). Eight of 11 patients received postoperative chemotherapy, and the mean survival period after the surgery was at least 14.1 months (2–36 months). However, of the 3 patients who did not receive combination therapy, 2 patients (17%) died 2 weeks after the surgery, and the survival time of the remaining one was unknown. This suggests that aggressive surgery combined with adjuvant chemotherapy may extend the survival more considerably compared with the administration of only TURBT and chemotherapy.

**Table 1:** Reported cases of primary bladder PNET.

References	Age/sex	Symptoms	Risk factor	Diagnostic	Tumor size	Metastasis	Surgery	further treatment	Survival
Banerjee et al^[20]^	M/21	Frequency, dysuria, hematuria	Drug, Renal transplant	IVP, cystoscopy	8 × 6 × 4 cm	None	cystectomy	Chemotherapy (VAC)	At least 18 mo
Gousse et al^[24]^	F/15	hematuria	None	IVP, cystoscopy	3 × 2 × 2 cm	None	TURBT	Chemotherapy (VAC + IE)	At least 18 mo
Desai^[21]^	F/38	Hematuria	HL	Cystoscope biopsy	12 × 7.0 × 3.5 cm	None	Cystectomy + TH + BSO	–	–
Mentzel et al^[8]^	M/62	Dark urine, fever, backache, AUR	Anemia	MRI	14 × 10 × 10 cm	Rectal and retroperitoneal tissue	TURBT + Nephrostomy	None	Died 2 wks later
Colecchia et al, 2002^[14]^	F/61	Hydronephrosis, renal failure	Diabetes, hypertension, IHD, thalassemia	CT, Cystoscope biopsy	–	pulmonary	–	–	–
Kruger et al^[9]^	M/81	Lymphedema, fatigue, urge incontinence, hydronephrosis	None	US, CT	–	Pelvic and retroperitoneal tissue	TURBT + Nephrostomy	None	Died 2 wks later
Ellinger et al^[15]^	M/72	Hematuria, oliguria	chemotherapy	MRI	–	Frozen pelvis, ileum	TURBT	–	At least 2 mos
Lopez-beltran et al^[22]^	F/21	Frequency, dysuria, hematuria	None	US, Cystoscope biopsy	9 × 8 × 6 cm	None	Cystectomy + TH + BSO	Chemotherapy + Imatinib	At least 36 mo
Osone et al^[6]^	M/10	Dysuria, hematuria	ALL, chemotherapy	US, CT, Cystoscope	1cm	None	TURBT	Chemotherapy (CDV + IE)	At least 2 yrs
Al Meshaan et al^[10]^	F/67	Hematuria, fever, hydronephrosis	Diabetes, hypertension, SCC of urinary bladder	US, CT, Cystoscope	3.0 × 2.5 × 1.0 cm	Pelvic lymph, pulmonary	TURBT + partial cystectomy	Chemotherapy	Died 8 mo later
Rao et al^[3]^	F/14	Dull Pain, lower-abdominal lump	None	US, CT, needle biopsy	15 × 12 × 7.5 cm	None	partial cystectomy	Chemotherapy	At least 6 mo
Busato et al^[16]^	F/52	Frequency, dysuria, pelvic pain, hematuria	None	US, Cystoscope	3.3 × 1.5 × 2.2 cm	None	TURBT	Chemotherapy (VAC + IE)	At least 27 mo
Okada et al^[11]^	M/65	Hematuria, dysuria	Hypertension, IHD	US, CT, Cystoscope	5 cm	pulmonary	TURBT + cystectomy	Chemotherapy (VIDE) + radiotherapy	Died 22 mo later
Zheng et al^[12]^	M/74	Frequency, dysuria, hematuria	None	CT	–	None	TURBT	Chemotherapy (VAC)	Died 4 mo later
Sueyoshi et al^[25]^	M/10	Polyuria, lower-abdominal swelling	None	US, CT	13.5 × 13.1 × 12.9 cm	None	Double J tube + partial cystectomy	Chemotherapy (VAC + IE)	At least 11 mo
Lam et al^[23]^	F/30	Polyuria, hematuria	None	US, MRI	6.4 × 9.4 × 7.7 cm	None	TURBT + cystectomy + indiana pouch	Chemotherapy (VAC + IE)	–
Tonyali et al^[4]^	F/38	Hematuria	None	CT	4 × 2.6 × 2.5 cm	None	TURBT + cystectomy + TH + BSO + ileal conduit	Chemotherapy (VAC + IE)	At least 14 mo
Vallonthaiel et al^[16]^	F/27	Frequency, hematuria,	Hyperparathyroidism	US, CT	10.3 × 9.8 × 4.7 cm	Pelvic lymph node	TURBT	Chemotherapy (VAC)	At least 3 mo
Present case	F/45	Frequency, urgency, dysuria	None	US, CT, Cystoscope	3 cm	None	TURBT + cystectomy + TH + ileal conduit	Chemotherapy (VAC)	At least 24 mo

Whether TURBT of PNET of the bladder is an effective therapy is unclear. In our case, comprehensive treatment was particularly effective. So, we recommend that early laparoscopic radical cystectomy and standard lymph node dissection combined with postoperative chemotherapy regimens may be essential to improving the prognosis of urinary bladder PNET.

## Acknowledgments

We would like to thank Jie Chen of the Department of Urology, Jiangxi Provincial People's Hospital Affiliated to Nanchang University for their operation on the patient.

## Author contributions

**Acquisition of data:** L.G, G.M.H, and H.Y.J.

**Analysis and Interpretation of date:** L.G, J.C, W.J.X, K.L, G.M.H, and Y.K.O.

**Conceptualization:** Win-Jie Xie, Jie Chen.

**Data curation:** Win-Jie Xie, Yuanhai Ji.

**Drafting of the Manuscript:** L.G, J.C, and W.J.X.

**Formal analysis:** Gao-Min Huang, Yuanhai Ji, Yangkang Ou, Jie Chen.

**Investigation:** Win-Jie Xie, Kun Li, Gao-Min Huang.

**Methodology:** Kun Li, Gao-Min Huang, Yangkang Ou.

**Project administration:** Kun Li.

**Resources:** Win-Jie Xie, Kun Li, Gao-Min Huang, Yuanhai Ji.

**Software:** Gao-Min Huang.

**Study conception:** J.C, L.G.

**Study design:** J.C, L.G, and W.J.X.

**Writing – original draft:** Liang Gao.

**Writing – review & editing:** Liang Gao, Kun Li, Jie Chen.

All authors have contributed to the critical revision and approval of the final manuscript.
